# Sera of Healthy First-Degree Relatives of SLE Patients Contain Autoantibodies to Globular Domains of C1q

**DOI:** 10.3390/antib15040062

**Published:** 2026-07-20

**Authors:** Ginka Cholakova, Alexandra Atanasova, Alexandra Kapogianni, Dobroslav Kyurkchiev, Bogdan Penev, Ivanka Tsacheva

**Affiliations:** 1 Faculty of Biology, Department of Biochemistry, Sofia University, 1164 Sofia, Bulgaria; alexandra.anatolieva@gmail.com (A.A.); kapogianni@uni-sofia.bg (A.K.); itsacheva@biofac.uni-sofia.bg (I.T.); 2 Laboratory of Clinical Immunology, University Hospital “St. Ivan Rilski”, Department of Clinical Immunology, Medical University of Sofia, 1431 Sofia, Bulgaria; dkyurkchiev@medfac.mu-sofia.bg; 3 Rheumatology Department, Sofiamed University Hospital, 1336 Sofia, Bulgaria; bogdanfpenev@gmail.com

**Keywords:** first-degree relatives, SLE, C1q, C3, Factor H, PLA2 activity

## Abstract

Background/Objectives: The autoimmune disorder Systemic Lupus Erythematosus (SLE) is characterized by increased titers of autoantibodies with different specificities against autoantigens, including the complement proteins C1q, C3 and Factor H. SLE is characterized by chronic inflammation and tissue damage due to the secretion of pro-inflammatory molecules and a tissue deposition of immune complexes formed by autoantibodies and their target antigens. The inflammatory process in SLE is maintained by the phospholipase A2 (PLA2) enzymes generating pro-inflammatory lipid mediators. Hereditary and environmental factors trigger SLE, with increased genetic heritability in first-degree relatives of SLE patients. Methods: A cohort of 48 healthy FDRs of SLE patients was analyzed with the ELISA method for the presence of antibodies to complement proteins C1q, C3 and Factor H, with a focus on detecting autoepitopes both on immobilized and soluble C1q and its globular fragments ghA, ghB and ghC. The total serum PLA2 activity of FDRs was measured using the chromogenic substrate 4-nitro-3-octanoyloxy-benzoic acid (NOBA). Results: Only C1q, and specifically its globular domains in both an immobilized and soluble state, was targeted by antibodies in the healthy FDRs similarly to the pattern established in SLE patients. In contrast, C3 and Factor H which are known autoantigens in SLE were not found as targets for the antibodies in the analyzed FDRs. Some of the FDRs showed increased serum PLA2 activity, which correlated weakly with anti-C1q antibodies. Conclusions: C1q and its globular domains are estimated as autoantigenic molecules for binding in the analyzed FDRs of SLE patients.

## 1. Introduction

Systemic Lupus Erythematosus (SLE) is an autoimmune disorder characterized by chronic tissue inflammation and its development involves epigenetic, genetic, ecological, and environmental factors [1]. It is characterized by the generation of a diverse set of autoantibodies that specifically bind to double-stranded DNA [2,3], nuclear fragments [4,5], ribosomal proteins [6,7], phospholipids [8,9] and complement proteins [10]. The most commonly detected autoantibodies against complement proteins are anti-C1q antibodies, which are found in approximately one third of patients with SLE and they are an established serum biomarker for clinical evaluation of SLE [11,12]. The first autoepitopes of C1q were mapped to the collagen-like region of C1q (anti-CLR antibodies). Later the globular domain of C1q, composed of three types of globular fragments A, B and C (anti-gC1q antibodies), was also found to be autoantigenic [13]. A recent study revealed that not only the conformation of the immobilized C1q displayed autoepitopes but also the conformation of the soluble C1q, and more specifically gC1q, was recognized by lupus antibodies [14]. Despite numerous studies, the main molecular mechanisms that trigger development of SLE remain unknown. There is mounting evidence that both hereditary and environmental factors are involved. Multiple research studies have consistently showed that the genetic heritability of SLE development is increased in the first-degree relatives (FDRs) of patients with SLE [15,16,17,18]. FDRs of SLE patients often exhibit subclinical immune dysregulation, including elevated autoantibodies, interferon signatures, and cytokine imbalances, positioning them as an at-risk group for potential progression to clinical disease [19,20]. The generation of anti-complement antibodies, particularly anti-C1q antibodies, exhibit familial clustering in families affected by SLE, underlying the genetic risk factor for their production even in unaffected individuals [13,21]. It has been reported that autoantibodies develop several years before the clinical onset of SLE [22], which makes healthy FDRs of SLE patients a valuable target group for investigation.

Many autoimmune disorders, including SLE, are based on chronic inflammation which involves the participation of phospholipase enzymes. The phospholipase A2 enzymes (PLA2) are most notable for their ability to hydrolyze membrane phospholipids thereby promoting the generation of proinflammatory lipid mediators [23].

The complement proteins C3 and Factor H (FH) are also targeted by SLE autoantibodies. In our recent research we found that approximately 30% of a Bulgarian cohort of SLE patients had anti-C3 antibodies and approximately 20% had anti-FH [24]. The anti-C3 autoantibodies tended to have lower affinity compared to the rarely occurring anti-FH and the more frequent anti-C1q antibodies. We also observed a weak correlation between anti-C3 antibodies and the serum PLA2 activity in SLE patients, whereas the correlation between anti-C3 and anti-FH autoantibodies was moderate.

In the present study, we analyzed a cohort of healthy FDRs of SLE patients for the presence of antibodies against the complement proteins C1q, C3 and Factor H, with a particular focus on detecting autoepitopes on both immobilized and soluble C1q and its globular fragments ghA, ghB and ghC. We also analyzed the association of each type of autoantibodies and the serum PLA2 activity of FDRs.

## 2. Materials and Methods

### 2.1. Study Population

- Group of healthy FDRs of SLE patients

Serum samples from 48 clinically healthy FDRs of SLE patients were enrolled in this study. The clinical parameters of FDRs were provided by Penev et al., 2023 [20]. The healthy FDR group had a median age of 43 years (range 19–71 years) and a female to male ratio of 7:1.

B. Group of SLE patients

Serum samples from 48 patients with SLE, previously analyzed [14], were used for comparison.

C. Control group

Pooled sera from 26 healthy donors were used as a control. Serum samples were provided from Aleksandrovska University Hospital of Sofia Medical University.

All sera were stored at −30 °C before analysis.

The study was conducted according to the guidelines of the Declaration of Helsinki and approved by the Ethics Committee of Sofia University.

### 2.2. Buffers

(a) ELISA buffers: phosphate-buffered saline (PBS): 0.01 M Na_2_HPO_4_, 0.01 M NaH_2_PO_4_, and 0.145 M NaCl, at pH 7.2; PBS, containing 0.05% Tween-20 (TPBS); PBS, containing 0.75 M NaCl (PBS/0.75); Carbonate buffer (CB): 100 mM NaHCO_3_ and 100 mM Na_2_CO_3_, at pH 9.6; alkaline phosphatase buffer (AP): 100 mM Tris, 100 mM NaCl, and 5 mM MgCl_2_, at pH 9.6.
(b) Buffer for phospholipase (PLA2) activity: 50 mM Tris-HCl, 10 mM CaCl_2_, and 50 mM NaCl, at pH 8.0; substrate–NOBA (4-Nitro-3-(octanoyloxy) benzoic acid (Enzo Life Sciences, Farmingdale, NY, USA).
(c) Buffers for extraction and purification of expressed gC1q fragments ghA, ghB and ghC: Lysis buffer: 50 mM Tris-HCl, at pH 8.0, containing 0.5 M NaCl, 1 mM EDTA, 1 mM Benzamidine-HCl, 0.25% Tween-20, 0.25% Triton-x100, 0.25% NP-40, and 100 μg/mL lysozyme; dialysis buffer: 20 mM Tris-HCl, at pH 8.0, containing 0.1 M NaCl, 1 mM EDTA, 5% glycerol, and 0.25% Tween-20; Elution buffer: 20 mM Tris-HCl, at pH 8.0, containing 0.1 M NaCl and 10 mM maltose.

### 2.3. PLA2 Activity Assay

The serum samples of all analyzed study groups were centrifuged at 12,000 rpm for 10 min at 4 °C and measured spectrophotometrically at 280 nm to determine the total protein concentration. Next, the sera were diluted at a 1:100 ratio in PBS and incubated in 96-flat bottom microtiter plates with 25 μL of serum sample/well. Buffer for PLA2 activity was added at 220 μL/well. NOBA chromogenic substrate (1 mg/mL, diluted in acetonitrile) was added at 5 μL/well. The control wells without serum contained 5 μL of NOBA substrate solution and 220 μL of buffer for PLA2 enzyme activity. Another type of control wells were coated with pooled serum from 24 healthy donors and the wells were treated with the same reagents as the wells containing the SLE serum samples. The microtiter plate was incubated for 15 min at 37 °C and then measured at 450 nm with a microplate reader (Chromate 4300, Awareness Technology, Palm City, FL, USA). All samples were analyzed in triplicate. The PLA2 activity in tested sera samples was expressed as specific activity—IU/protein and mg/mL. The relative PLA2 activity was presented as a percentage following the equation:

PLA2 activity [%] = (PLA2 activity of tested serum sample)/(PLA2 activity of pooled sera from healthy donors) × 100

The measured triplicate values for each tested serum were averaged, and the respective standard deviation (SD) values were subtracted. The cut-off value was determined by calculating the sum of the averaged triplicate values for the healthy donors and the respective SD. The analyzed sera were considered positive for increased PLA2 activity if they exceeded the cut-off.

### 2.4. Growth Media

The following growth media were used: 2xTY medium (20 g/L Tryptone, 10 g/L Yeast Extract, 20 g/L NaCl, pH 7.2) containing 100 μg/mL ampicillin and supplemented with 1 mM MgSO_4_ and 1% glucose; Luria–Bertani (LB) medium (10 g/L Tryptone, 5 g/L Yeast Extract, 10 g/L NaCl, pH 7.2), containing 100 μg/mL ampicillin and supplemented with 1 mM MgSO_4_ and 1% glucose; ZYP-5052 medium for autoinduction (1% Tryptone, 0.5% Yeast Extract, 25 mM (NH_4_)_2_SO_4_, 50 mM KH_2_PO_4_, 50 mM Na_2_HPO_4_, 0.5% glycerol, 0.05% glucose, 0.2% α-lactose, 1 mM MgSO_4_) containing 100 μg/mL ampicillin; and 5xM9 medium (33.9 g/L Na_2_HPO_4_, 15 g/L NaH_2_PO_4_, 2.5 g/L NaCl, 5 g/L NH_4_Cl) with minimal M9 agar medium (1xM9, 0.2 mM MgSO_4_, 0.1 mM CaCl_2_, 0.2% glucose and 1.5% agar).

### 2.5. Expression and Purification of Recombinant gC1q Fragments

Expression of the recombinant gC1q fragments ghA, ghB and ghC was induced by a combined approach of induction with 0.5 mM IPTG for 3 h at 25 °C and by overnight autoinduction at 25 °C. Briefly, the gC1q fragments were induced as fusion proteins with maltose-binding protein (MBP) in the bacterial strain *E. coli* BL21 transformed by plasmids pKBM/A or pKBM/B or pKBM/C, respectively [25]. The cells were grown on minimal M9 agar plates and then cultivated in LB to OD600 = 0.9 for IPTG induction or cultivated in ZYP-5052 for 18–24 h. The induced *E. coli* cells were lysed in Lysis buffer for gC1q for 15 min on ice. The lysed cells were centrifuged at 9000 rpm for 45 min at 4 °C. The supernatant containing the recombinant globular heads was dialyzed against the dialysis buffer for gC1q overnight, filtered through a 0.45 μm PES membrane filter and subjected to amylose affinity chromatography. The bound proteins were eluted with Elution buffer for gC1q. The eluted protein samples were dialyzed against PBS overnight and their concentration and quality were analyzed with estimated absorbance measured at 280 nm and with 4–16% SDS-PAGE gel electrophoresis.

### 2.6. Biotin Labeling

The human C1q (Merck Millipore Calbiochem™, Darmstadt, Germany) and the purified recombinant globular fragments ghA, ghB and ghC were dialyzed in dialysis tubing (SpectraPor^®^, Repligen, Rancho Dominguez, CA, USA) against PBS, at pH 8.5, overnight followed by the addition of biotin (D(+)Biotin N-hydroxysuccinimide ester, Acros Organics, Geel, Belgium) prepared as a stock solution in distilled water as 10 μg/μL. The added biotin was added as 1/6th of the amount of tested protein samples and the biotin incubation was performed at 25 °C for 4 h with low-speed shaking. After incubation, the biotinylated complement proteins were dialyzed against PBS, at pH 7.2, overnight at 4 °C.

### 2.7. Enzyme-Linked Immunosorbent Assay (ELISA)

In all ELISA experiments the plates were washed three times with TPBS (200 μL/well) after each incubation of the reagent. The blocking step was performed with 1% BSA (200 μL/well) for 1 h at 37 °C.

(a) Healthy donors: Polystyrene 96-flat bottom well plates were coated with C1q (2 μg/well) for 1 h at 37 °C. The wells were blocked and incubated with sera samples of 26 clinically healthy people (1:100 diluted in PBS/0.75) overnight at 4 °C. After washing, rabbit anti-human IgG conjugated with alkaline phosphatase (Dako, Glostrup, Denmark), diluted 1:2000 in TPBS, was incubated for 1 h at 37 °C. The bound complexes were detected by 0.5 mg/mL pNPP (Acros Organic) dissolved in AP buffer and the absorbance at 405 nm was read on a Chromate 4300 Microplate reader (Awareness Technology, USA).
(b) Immobilized C1q and gC1q: Polystyrene 96-flat bottom well plates were coated with 2 μg/well of the tested proteins: C1q, ghA, ghB and ghC, dissolved in CB for 1 h at 37 °C. Next, the plates were blocked and incubated with tested human sera (1:100 diluted in PBS/0.75) overnight at 4 °C. After washing, rabbit anti-human IgG conjugated with alkaline phosphatase (Dako), diluted 1:2000 in TPBS, was incubated for 1 h at 37 °C. The bound complexes were detected as described above.
(c) Soluble C1q and gC1q: The plates were coated with tested human sera (1:50 diluted in CB, 100 μL/well) overnight at 4 °C. The plates were blocked and incubated with one of the following biotinylated proteins overnight at 4 °C: C1q, ghA, ghB, ghC (2 μg/well in PBS/0.75). Next, Extravidin-AP (Sigma-Aldrich, St. Louis, MO, USA) diluted 1:100,000 in TPBS was incubated for 1 h at 37 °C. The bound complexes were detected by 0.5 mg/mL pNPP (Acros Organic) dissolved in AP buffer and the absorbance at 405 nm was read on the Chromate 4300 Microplate reader (Awareness Technology, USA).
(d) Immobilized C3: Polystyrene 96-flat bottom well plates were coated with 1 μg/well of human C3 (QuidelOrtho, San Diego, CA, USA) dissolved in CB for 1 h at 37 °C. The plates were blocked and incubated with tested human sera (1:100 diluted in PBS) overnight at 4 °C. After washing, rabbit anti-human IgG conjugated with alkaline phosphatase (Dako), diluted 1:2000 in TPBS, was incubated for 1 h at 37 °C. The bound complexes were detected as described above.
(e) Immobilized Factor H: Polystyrene 96-flat bottom well plates were coated with 1 μg/well of human Factor H (QuidelOrtho, San Diego, CA, USA) dissolved in CB for 1 h at 37 °C. The plates were blocked and incubated with tested human sera (1:100 diluted in PBS) overnight at 4 °C. After washing, rabbit anti-human IgG conjugated with alkaline phosphatase (Dako), diluted 1:2000 in TPBS, was incubated for 1 h at 37 °C. The bound complexes were detected as described above.

### 2.8. Statistical Analysis

Statistical analysis of results was performed in GraphPad Prism software version 8.0.1. The Mann–Whitney U test for continuous variables for 2-group comparisons was used. Quantitative data were expressed as mean ± SD. The Pearson correlation was used to analyze correlation. Statistical significance was considered as *p* < 0.05.

## 3. Results

We performed a multitarget study of healthy FDRs, testing their serum samples for quantitation of antibodies to three complement autoantigens—C1q, C3 and FH. The autoantigenic behavior of C1q was analyzed in two settings—in an immobilized state and in a soluble state referring to both the intact protein and its gC1q, represented by ghA, ghB and ghC. Since IgG of any specificity is a ligand to C1q via the Fcγ region, all analyses with this antigen were carried out under high-ionic-strength conditions so that IgG antibodies were bound to C1q by their Fab only. As a control we used 24 clinically healthy donors who were not related to SLE patients. In order to assess the contribution of anti-C1q antibodies as a manifestation of a genetic predisposition, we compared the FDRs titers with the titers of 48 patients with active SLE. The average of the measured values for the healthy donors was used as a reference to determine positive sera for the presence of autoantibodies that bind to tested autoantigens. Analyzed SLE sera samples and FDR sera samples were considered positive for autoantibodies against C1q and its globular fragments if they exceeded the control value plus three times its standard deviation (average sample—SD sample > average control + 3 × SD control).

**Figure 1:**
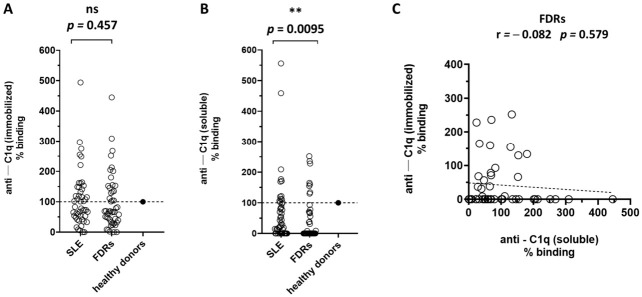
Presence of anti-C1q autoantibodies against immobilized and soluble C1q in sera of FDRs (n = 48) and SLE patients (n = 48). (**A**) Binding (%) of autoantibodies against immobilized C1q; (**B**) binding (%) of autoantibodies against soluble C1q. One dot = 1 serum; dashed line: normal cut-off trendline represented as a 100% binding (%) of antibodies presented in a pooled serum from 26 clinically healthy donors against immobilized or soluble C1q; (**C**) Pearson correlation analysis of autoantibodies against immobilized and soluble C1q in FDRs. Statistical analysis was determined with Mann–Whitney U test; ns: not significant; ** (*p* ≤ 0.01).

A total of 48 FDRs compared to 48 SLE patients were analyzed for the presence of autoantibodies against immobilized and soluble human C1q, ghA, ghB and ghC. We found that 37.50% (18/48) of tested FDRs compared to 31.25% of SLE patients (15/48) were positive for anti-C1q autoantibodies against immobilized C1q (Figure 1A). The frequencies of positive sera with anti-soluble C1q autoantibodies were 16.66% (8/48) among the FDRs and 18.75% (9/48) among the SLE patients (Figure 1B). Among the FDRs, the autoantibodies to immobilized C1q negatively correlated with those to soluble C1q (r = −0.082, *p* = 0.579) (Figure 1C).

**Figure 2:**
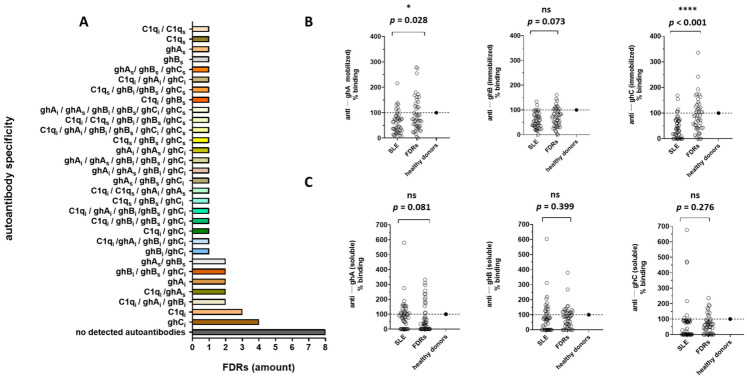
Autoantibody specificities against immobilized and soluble C1q and its globular fragments ghA, ghB and ghC in FDR sera (n = 48) and SLE patients (n = 48). (**A**) Estimated autoantibody specificities against immobilized (i) and soluble (s) C1q and its globular fragments ghA, ghB and ghC in the tested cohort of FDRs; (**B**) binding affinity (%) of autoantibodies against immobilized ghA (**left**), ghB (**middle**), and ghC (**right**) in tested FDRs and SLE patients. (**C**) Binding affinity (%) of autoantibodies against soluble ghA (**left**), ghB (**middle**), and ghC (**right**) in tested FDRs and SLE patients. One dot = 1 serum; dashed line: normal cut-off trendline represented as a 100% binding affinity (%) of antibodies presented in a pooled serum from 26 clinically healthy donors against immobilized or soluble C1q. Statistical analysis for panels (**B**,**C** was determined with Mann–Whitney U test; ns: not significant; * (*p* ≤ 0.05), **** (*p* ≤ 0.001).

In order to estimate whether part of the FDRs anti-C1q antibodies were specific for epitopes of the gC1q, we performed an ELISA analysis using recombinant ghA, ghB and ghC as antigens. Data revealed that most of the FDRs were positive for a mixture of autoantibodies against immobilized and/or soluble C1q and/or its globular fragments (Figure 2A). In four FDRs autoantibodies were detected only against immobilized ghC (anti-ghCi). One FDR (1/48) was positive only for autoantibodies against immobilized and soluble C1q. Another eight (8/48, 16.66%) FDRs were not found positive for any type of tested autoantibodies. Positive FDRs for autoantibodies against immobilized gC1q fragments were as follows: 27.08% of FDRs (13/48) were positive for anti-ghA antibodies compared to 16.66% of SLE patients (8/48), 29.17% of FDRs (14/48) were positive for anti-ghB antibodies compared to 8.33% of SLE patients (4/48) and 39.58% of FDRs (19/48) were positive for anti-ghC antibodies compared to 8.33% of SLE patients (4/48) (Figure 2B). According to the analyzed C1q-globular fragments as soluble autoantigenic molecules, we found that 25% of FDRs (12/48) were positive for anti-ghA autoantibodies compared to 33.33% of SLE patients (16/48), 35.42% of FDRs (17/48) were positive for anti-ghB autoantibodies compared to 31.25% of SLE patients (15/48) and 12.5% of FDRs (6/48) were positive for anti-ghC autoantibodies compared to 8.33% of SLE patients (4/48) (Figure 2C).

**Figure 3:**
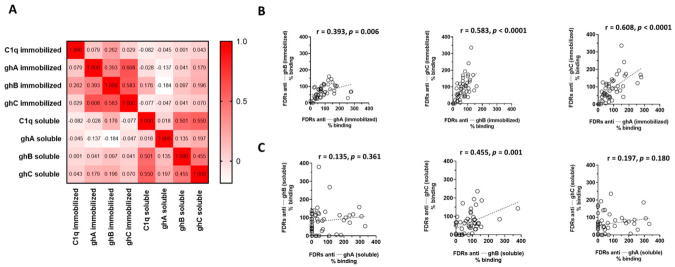
Correlation between the binding affinity (%) of autoantibodies against immobilized and soluble C1q and its globular fragments in FDRs. (**A**) Heatmap of r values obtained from Pearson correlation analysis. (**B**) Correlation of serum anti-gC1q antibodies against immobilized gC1q fragments; (**C**) correlation of serum anti-gC1q antibodies against soluble gC1q fragments.

We performed Pearson correlation analysis to determine the possible correlations between different autoantibodies against immobilized and soluble C1q and its globular fragments among the FDR cohort (Figure 3A). The correlation analysis of autoantibody specificities against immobilized globular fragments ghA, ghB and ghC revealed a weak correlation between anti-ghA and anti-ghB (r = 0.393, *p* = 0.006), a moderate correlation between anti-ghB and anti ghC (r = 0.583, *p* < 0.0001), and a strong correlation between anti-ghA and anti-ghC (r = 0.608, *p* < 0.0001) (Figure 3B). The correlation between autoantibodies against soluble globular fragments was weaker: anti-ghA and anti-ghB (r = 0.135, *p* = 0.361) and anti-ghA and anti-ghC (r = 0.197, *p* = 0.180), with moderate correlation among anti-ghB and anti-ghC (r = 0.492, *p* = 0.0004) (Figure 3C).

**Figure 4:**
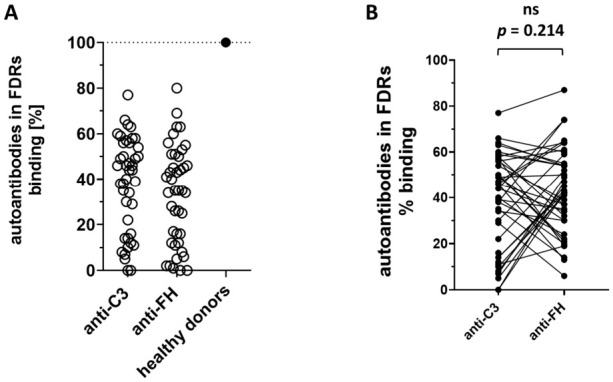
ELISA assay for the presence of autoantibodies against C3 and factor H in FDRs. (**A**): One dot = 1 serum sample; healthy donors: pooled sera sample from healthy donors (n = 26). The horizontal dotted line indicates the cut-off value; (**B**): comparative analysis of anti-C3 and anti-FH autoantibodies in individual sera of FDRs; ns by paired *t* test.

Next, we analyzed the FDR sera for the presence of antibodies to C3 and FH. None of the tested sera were found to be positive (Figure 4).

**Figure 5:**
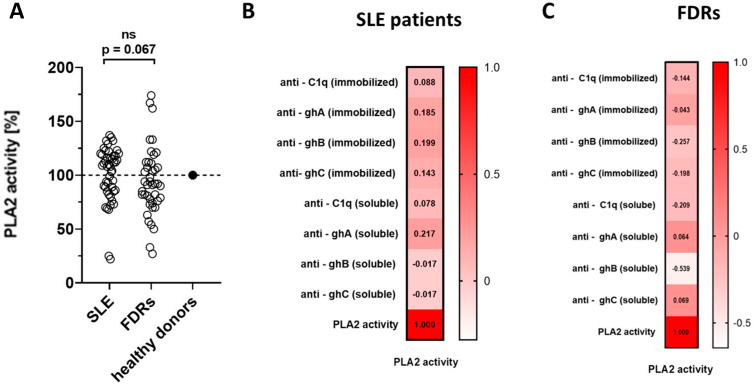
Measurement of PLA2 specific activity in sera samples of SLE patients and FDRs (**A**). One dot = 1 serum sample; healthy donors: pooled sera from healthy donors (n = 26). The horizontal dotted line indicates the cut-off value. Statistical analysis for panel (**A**) was determined with Mann–Whitney U test; ns: not significant. Heatmap with Pearson correlation coefficients for autoantibodies specific to C1q, ghA, ghB and ghC in SLE patients (**B**) and FDRs (**C**) with serum PLA2 activity.

Further, we analyzed the serum PLA2 activity in the SLE patients and the FDRs. Each sample was measured in triplicate and the values were averaged with the respective SD subtracted. The cut-off value was determined by calculating the sum of the averaged triplicate values for the healthy donors and the respective SD. The analyzed sera were considered positive for increased PLA2 activity if they exceeded the cut-off. The screening revealed that 26 out of 48 SLE patients (54.16%) were positive for increased serum PLA2 activity and 16 out of 48 FDRs (33.33%) were positive (Figure 5A). Since the FDRs were negative for anti-C3 and anti-FH, the correlation analysis between the serum PLA2 activity and the autoantibodies in SLE sera and FDR sera was performed only for C1q (Figure 5B,C). Generally, the anti-C1q and anti-gC1q antibodies showed a very weak correlation with serum PLA2 activity in the SLE group with the exception of the soluble ghB and ghC antigens which had negative correlation. In the FDR group all antigens showed a negative correlation with the only exception of the soluble ghA. Interestingly, the soluble ghA had the highest coefficient in the SLE group, although the correlation was weak.

## 4. Discussion

The SLE has been researched over a long period of time but some of the molecular aspects of the triggering mechanism are still unknown. There are clear clinical criteria for diagnosing the condition; the predisposition factors for developing SLE are established yet it is impossible to estimate whether or not an onset of SLE would be triggered. Therefore, it is of great value to know as much as possible about the molecular events, or rather the network of molecular events preceding a clinical manifestation of the autoimmune condition. The first-degree relatives of SLE patients are a target group of choice when the genetic contribution is to be assessed.

We had the chance to analyze a cohort of FDRs who rarely participate willingly in such projects. Recently we studied a Bulgarian cohort of SLE patients [14,24] that provided a valuable basis for comparison with the FDRs [20]. We analyzed whether three complement proteins, namely C1q, C3 and Factor H, as known SLE autoantigens, were targeted by FDR antibodies. FDRs were found positive only for the antigen C1q (Figure 2 and Figure 4). C3 and FH were not recognized as autoantigens. The protein conformation of C1q was tested in two different states, immobilized and soluble, due to the recently discovered fact that the conformation of the soluble protein presents epitopes for the lupus autoantibodies [14]. The majority of physiological functions of C1q are carried out by its gC1q, so the binding of anti-gC1q could cause a functional deficiency, an estimated contributing factor for developing SLE [26,27,28,29]. Consequently, we included the globular fragments ghA, ghB and ghC in the analysis, which constitute one gC1q, also in immobilized and in soluble conformations.

Anti-C1q antibodies displayed a similar pattern, which we have found in the SLE patients. Firstly, both immobilized and soluble conformations of C1q and gC1q were antigenic (Figure 1 and Figure 2). Secondly, the two alternative conformations were recognized by different epitopes (Figure 3). Interestingly, the immobilized globular fragments ghA, ghB and ghC were recognized in more positive FDRs compared to SLE patients, while the soluble ghA, ghB and ghC showed similar degrees of antigenicity in the two analyzed cohorts (Table 1) [14].

Only ghC showed a different pattern in the comparison between FDRs and SLE patients. Unlike ghA and ghB, the immobilized state of ghC was the prevalent antigen among the positive FDRs, while in the SLE group the soluble states of ghA and ghB prevailed among the positive patients.

Among the positive FDRs the soluble and the immobilized conformations of ghA and ghB were antigenic to a similar extent suggesting that within a pre-clinical state the serum C1q is as much antigenic as the immobilized one. The strong correlation between the immobilized ghA and ghC as antigens among the positive FDRs (Figure 3B) corresponds to their combined autoantigenicity established for a Bulgarian Lupus Nephritis cohort [30]. The antigenic behavior of soluble C1q is marked by a moderate correlation with the soluble ghB implying that the intact protein in serum is mainly presented as an antigen by its ghB.

The weak, and even negative, correlation between the titers of anti-C1q and the increased serum activity of PLA2 in FDRs suggests that the activated macrophages and the autoantibodies contribute to the autoimmune microenvironment in different ways following distinct mechanisms. Notably, ghA is the antigen showing the highest correlation coefficient for the SLE patients, although a weak one, and at the same time ghA is the only antigen that is in a positive correlation with PLA2 activity for the FDRs. This might be related to the fact that ghA has been identified as a carrier of a cross-epitope with anti-dsDNA antibodies [31], which are among the first autoantibodies to appear in clinically manifested SLE and are one of the criteria to diagnose the disease.

The in-house ELISAs developed and employed in this study for the detection of autoantibodies against C1q and its recombinant globular head fragments ghA, ghB, and ghC, as well as C3 and Factor H, demonstrated several functional characteristics that support their reliability in the context of this research. These assays showed acceptable reproducibility and low background signals in negative controls. High-ionic-strength conditions were applied in the C1q-specific assays in order to inhibit the binding of C1q to CH2 domains of IgG molecules with irrelevant antigenic specificities according to previously reported ELISA assays. The antibody specificity was further supported by the differential reactivity observed against the individual globular fragments ghA, ghB, ghC, when compared to intact C1q, consistent with epitope-specific recognition reported in SLE. The in-house ELISAs used in our current study are comparable to previously reported and validated in-house and commercial ELISA assays for detection of anti-C1q [12,14], anti-C3 autoantibodies [32] and anti-Factor H autoantibodies [33]. Thus, our performed ELISA assays targeting the globular heads ghA, ghB, and ghC are based on experimental protocols that have been described previously and are considered important for dissecting epitope specificity in lupus nephritis and related conditions [34,35]. While commercial kits offer standardization advantages, the in-house format allowed us to simultaneously assess reactivity against multiple native and recombinant complement components using the same serum cohort under uniform conditions, which was particularly suited for exploring the immune status in first-degree relatives of SLE patients. Limitations include the lack of direct head-to-head comparison with all available commercial assays and the need for broader multi-center validation; nevertheless, the present assays provided consistent and biologically plausible results that warrant further investigation.

A notable aspect of anti-C1q antibodies is the differential recognition of C1q in its soluble versus immobilized (solid-phase) forms. It has been widely accepted that anti-C1q autoantibodies in SLE patients often bind more strongly or preferentially to immobilized C1q, likely due to the exposure of cryptic epitopes upon surface binding or incorporation into immune complexes, while recognition of soluble C1q is less frequent or weaker in some patients [36,37,38]. However, in our present study and in another previous study, we assessed the presence of anti-C1q antibodies against soluble (non-immobilized) C1q [14]. Whether first-degree relatives (FDRs) exhibit a different pattern of reactivity toward soluble versus immobilized C1q compared to SLE patients warrants further investigation. It is plausible that the autoantibodies detected in FDRs reflect an early, non-pathogenic stage of autoimmunity that requires a “second hit”—such as environmental triggers, infections, or additional breaks in immune tolerance—for progression to clinical SLE and organ damage. This two-hit hypothesis may help explain why only a minority of autoantibody-positive FDRs ultimately develop an active disease.

The detection of autoantibodies against both immobilized and soluble C1q and its globular fragments in first-degree relatives of SLE patients holds potential clinical relevance. These autoantibodies are strongly associated with lupus nephritis and disease flares in established SLE. Their presence in FDRs may therefore serve as an early immunological marker of increased risk, enabling clinicians to identify individuals who could benefit from closer monitoring, lifestyle modifications, or preventive strategies before overt clinical disease develops. Including such autoantibodies into risk assessment for high-risk families could improve early detection and personalized treatment, ultimately contributing to better long-term outcomes.

From a broader perspective, it is well established that although a significant percentage of FDRs display autoantibodies and other shared immunological dysregulations, a much smaller percentage will develop overt SLE. As elegantly demonstrated by Munroe et al. [17], the defining factor in this process is the balance between proinflammatory and regulatory cytokines.

## 5. Conclusions

The comparison of the FDRs with clinically overt SLE patients outlines the importance of the complement protein C1q for the development of SLE. The conformations of both the immobilized and the soluble C1q present epitopes for autoantibodies which are likely to contribute to triggering of SLE by causing a functional C1q deficiency. Our results showed that many FDRs may represent a state of persistent but “benign” autoimmunity rather than impending clinical SLE. Longitudinal follow-up studies will therefore be essential to clarify the clinical significance of these autoantibodies. Larger prospective studies are required to validate our observations and fully elucidate the role of anti-complement autoantibodies in the immune status and disease risk of first-degree relatives of SLE patients.

## Figures and Tables

**Table 1:** Number of positive sera in the analyzed cohorts for anti-gC1q in immobilized and soluble state.

Autoantigen	FDR, n = 48	SLE, n = 48
ghAi	13	8
ghAs	12	16
ghBi	14	4
ghBs	17	15
ghCi	19	4
ghCs	6	4

## Data Availability

The original contributions presented in this study are included in the article material. Further inquiries can be directed to the corresponding author.

## Abbreviations

The following abbreviations are used in this manuscript:

FDRs	First-degree relatives
SLE	Systemic Lupus Erythematosus
PLA2	Phospholipase A2
NOBA	4-nitro-3-octanoyloxy-benzoic acid
FH	Factor H
C3	Complement component 3
CLR	collagen-like region
gC1q	globular head domains of C1q
ghA	globular head fragment A
ghB	globular head fragment B
ghC	globular head fragment C
ELISA	Enzyme-Linked Immunosorbent Assay
TPBS	Phosphate-buffered saline with 0.05% Tween-20
PBS	Phosphate-buffered saline
AP	Alkaline Phosphatase
CB	Carbonate buffer
EDTA	Ethylenediaminetetraacetic acid
Na_2_HPO_4_	Sodium phosphate dibasic
(NH_4_)_2_SO_4_	Ammonium sulfate
NaH_2_PO_4_	Sodium phosphate monobasic
KH_2_PO_4_	Potassium dihydrogen phosphate
NaCl	Sodium chloride
MgSO_4_	magnesium sulfate
HCl	Hydrochloric Acid
Tris-HCl	Tris(hydroxymethyl)aminomethane with hydrochloric acid
NP-40	Nonidet P-40
TY	Yeast Extract Tryptone medium
LB	Luria–Bertani medium
MBP	maltose-binding protein
E. coli	Escherichia coli
IPTG	Isopropyl β-d-1-thiogalactopyranoside
PES	polyethersulfone membrane
pNPP	para-Nitrophenylphosphate
SD	standard deviation
SDS-PAGE	Sodium Dodecyl Sulfate Polyacrylamide Gel Electrophoresis

## References

[1] Dai X., Fan Y., Zhao X. (2025). Systemic lupus erythematosus: Updated insights on the pathogenesis, diagnosis, prevention and therapeutics. Signal Transduct. Target. Ther..

[2] Wang X., Xia Y. (2019). Anti-double Stranded DNA Antibodies: Origin, Pathogenicity, and Targeted Therapies. Front. Immunol..

[3] Kubota T. (2023). An Emerging Role for Anti-DNA Antibodies in Systemic Lupus Erythematosus. Int. J. Mol. Sci..

[4] Al-Mughales J.A. (2022). Anti-Nuclear Antibodies Patterns in Patients With Systemic Lupus Erythematosus and Their Correlation with Other Diagnostic Immunological Parameters. Front. Immunol..

[5] Pisetsky D.S., Lipsky P.E. (2020). New insights into the role of antinuclear antibodies in systemic lupus erythematosus. Nat. Rev. Rheumatol..

[6] Kamstrup S.L., Schmidt N.S., Langkilde H.Z., Nilsson A.C., Voss A. (2025). Anti-ribosomal-P protein antibodies and systemic lupus erythematosus (SLE): In a cross-sectional study of Danish adult patients with SLE, no significant association is found between anti-ribosomal-P and neuropsychiatric SLE. Lupus Sci. Med..

[7] Caponi L., Giordano A., Bartoloni E.B., Gerli R. (2003). Detection of anti-ribosome antibodies: A long story of lights and shadows. Clin. Exp. Rheumatol..

[8] Richter P., Badescu M.C., Rezus C., Ouatu A., Dima N., Popescu D., Burlui A.M., Bratoiu I., Mihai I.R., Rezus E. (2024). Antiphospholipid Antibodies as Key Players in Systemic Lupus Erythematosus: The Relationship with Cytokines and Immune Dysregulation. Int. J. Mol. Sci..

[9] Ünlü O., Zuily S., Erkan D. (2016). The clinical significance of antiphospholipid antibodies in systemic lupus erythematosus. Eur. J. Rheumatol..

[10] Ayano M., Horiuchi T. (2023). Complement as a Biomarker for Systemic Lupus Erythematosus. Biomolecules.

[11] Trendelenburg M. (2021). Autoantibodies against complement component C1q in systemic lupus erythematosus. Clin. Transl. Immunol..

[12] Stojan G., Petri M. (2016). Anti-C1q in systemic lupus erythematosus. Lupus.

[13] Mahler M., van Schaarenburg R.A., Trouw L.A. (2013). Anti-C1q autoantibodies, novel tests, and clinical consequences. Front. Immunol..

[14] Atanasova A.A., Cholakova G.I., Kapogianni A.P., Donev V., Ivanova D., Yordanova A.D., Bogoeva V.P., Tsacheva I.G. (2025). C1q Is Recognized as a Soluble Autoantigen by Anti-C1q Antibodies of Patients with Systemic Lupus Erythematosus. Antibodies.

[15] Eroglu G.E., Kohler P.F. (2002). Familial systemic lupus erythematosus: The role of genetic and environmental factors. Ann. Rheum. Dis..

[16] Al Dhaheri A., Alblooshi H., Bharathan A., Alneyadi A., Al Ali M., Alzaabi A., Trad J., Nair S.C., Aljaberi N. (2025). The impact of familial autoimmunity and familial lupus on the clinical presentations and disease outcomes of SLE patients in the United Arab Emirates. BMC Rheumatol..

[17] Munroe M.E., Young K.A., Guthridge J.M., Kamen D.L., Gilkeson G.S., Weisman M.H., Ishimori M.L., Wallace D.J., Karp D.R., Harley J.B. (2022). Pre-Clinical Autoimmunity in Lupus Relatives: Self-Reported Questionnaires and Immune Dysregulation Distinguish Relatives Who Develop Incomplete or Classified Lupus From Clinically Unaffected Relatives and Unaffected, Unrelated Individuals. Front. Immunol..

[18] Kuo C.F., Grainge M.J., Valdes A.M., See L.C., Luo S.F., Yu K.H., Zhang W., Doherty M. (2015). Familial Aggregation of Systemic Lupus Erythematosus and Coaggregation of Autoimmune Diseases in Affected Families. JAMA Intern. Med..

[19] Alarcón-Segovia D., Alarcón-Riquelme M.E., Cardiel M.H., Caeiro F., Massardo L., Villa A.R., Pons-Estel B.A. (2005). Grupo Latinoamericano de Estudio del Lupus Eritematoso (GLADEL). Familial aggregation of systemic lupus erythematosus, rheumatoid arthritis, and other autoimmune diseases in 1,177 lupus patients from the GLADEL cohort. Arthritis Rheum..

[20] Penev B., Vasilev G., Todorova E.I., Tumangelova-Yuzeir K., Kurteva E., Monov S., Kyurkchiev D. (2023). First-degree relatives of patients with systemic lupus erythematosus: Autoreactivity but not autoimmunity?. Int. J. Rheum. Dis..

[21] Hunnangkul S., Nitsch D., Rhodes B., Chadha S., Roberton C.A., Pessôa-Lopes P., Norsworthy P.J., Fernando M.M., Charles P., Mackworth-Young C. (2008). Familial clustering of non-nuclear autoantibodies and C3 and C4 complement components in systemic lupus erythematosus. Arthritis Rheum..

[22] Arbuckle M.R., McClain M.T., Rubertone M.V., Scofield R.H., Dennis G.J., James J.A., Harley J.B. (2003). Development of autoantibodies before the clinical onset of systemic lupus erythematosus. N. Engl. J. Med..

[23] Burke J.E., Dennis E.A. (2009). Phospholipase A2 structure/function, mechanism, and signaling. J. Lipid Res..

[24] Cholakova G., Stankova S., Kapogianni A., Gendzhova M., Ivanova D., Petrova S., Tsacheva I. (2024). A study of the correlation between phospholipase A2 enzyme activity and anti-complement antibodies in patients with systemic lupus erythematosus. Pharmacia.

[25] Kishore U., Gupta S.K., Perdikoulis M.V., Kojouharova M.S., Urban B.C., Reid K.B. (2003). Modular organization of the carboxyl-terminal, globular head region of human C1q A, B, and C chains. J. Immunol..

[26] Kirschfink M., Petry F., Khirwadkar K., Wigand R., Kaltwasser J.P., Loos M. (1993). Complete functional C1q deficiency associated with systemic lupus erythematosus (SLE). Clin. Exp. Immunol..

[27] Lood C., Gullstrand B., Truedsson L., Olin A.I., Alm G.V., Rönnblom L., Sturfelt G., Eloranta M.L., Bengtsson A.A. (2009). C1q inhibits immune complex–induced interferon-α production in plasmacytoid dendritic cells: A novel link between C1q deficiency and systemic lupus erythematosus pathogenesis. Arthritis Rheum..

[28] Al-Mayouf S.M., Abanomi H., Eldali A. (2011). Impact of C1q deficiency on the severity and outcome of childhood systemic lupus erythematosus. Int. J. Rheum. Dis..

[29] Hussain K., Ladak S., Akbar F., Kirmani S., Altaf S. (2025). C1q deficiency with severe skin manifestations: A case report from Pakistan. Rare.

[30] Stoyanova V., Petrova S., Tchorbadjieva M., Deliyska B., Vasilev V., Tsacheva I. (2011). New insight into the autoimmunogenicity of the complement protein C1q. Mol. Immunol..

[31] Franchin G., Son M., Kim S.J., Ben-Zvi I., Zhang J., Diamond B. (2013). Anti-DNA antibodies cross-react with C1q. J. Autoimmun..

[32] Vasilev V.V., Noe R., Dragon-Durey M.A., Chauvet S., Lazarov V.J., Deliyska B.P., Fremeaux-Bacchi V., Dimitrov J.D., Roumenina L.T. (2015). Functional Characterization of Autoantibodies against Complement Component C3 in Patients with Lupus Nephritis. J. Biol. Chem..

[33] Dragon-Durey M.A., Loirat C., Cloarec S., Macher M.A., Blouin J., Nivet H., Weiss L., Fridman W.H., Frémeaux-Bacchi V. (2005). Anti-Factor H autoantibodies associated with atypical hemolytic uremic syndrome. J. Am. Soc. Nephrol..

[34] Moroni G., Trendelenburg M., Del Papa N., Quaglini S., Raschi E., Panzeri P., Testoni C., Tincani A., Banfi G., Balestrieri G. (2001). Anti-C1q antibodies may help in diagnosing a renal flare in lupus nephritis. Am. J. Kidney Dis..

[35] Trendelenburg M., Lopez-Trascasa M., Potlukova E., Moll S., Regenass S., Frémeaux-Bacchi V., Martinez-Ara J., Jancova E., Picazo M.L., Honsova E. (2006). High prevalence of anti-C1q antibodies in biopsy-proven active lupus nephritis. Nephrol. Dial. Transplant..

[36] Dijkstra D.J., van de Bovenkamp F.S., Abendstein L., Zuijderduijn R., Pool J., Kramer C.S.M., Slot L.M., Drijfhout J.W., de Vor L., Gelderman K.A. (2023). Human anti-C1q autoantibodies bind specifically to solid-phase C1q and enhance phagocytosis but not complement activation. Proc. Natl. Acad. Sci. USA.

[37] Pickering M.C., Botto M. (2010). Are anti-C1q antibodies different from other SLE autoantibodies?. Nat. Rev. Rheumatol..

[38] Calatroni M., Moroni G., Conte E., Stella M., Reggiani F., Ponticelli C. (2024). Anti-C1q antibodies: A biomarker for diagnosis and management of lupus nephritis. A narrative review. Front. Immunol..

